# RefSoil+: a Reference Database for Genes and Traits of Soil Plasmids

**DOI:** 10.1128/mSystems.00349-18

**Published:** 2019-02-26

**Authors:** Taylor K. Dunivin, Jinlyung Choi, Adina Howe, Ashley Shade

**Affiliations:** a Department of Microbiology and Molecular Genetics, Michigan State University, East Lansing, Michigan, USA; b Environmental and Integrative Toxicological Sciences, Michigan State University, East Lansing, Michigan, USA; c Department of Agricultural and Biosystems Engineering, Iowa State University, Ames, Iowa, USA; d Department of Plant, Soil and Microbial Sciences, Michigan State University, East Lansing, Michigan, USA; e Program in Ecology, Evolutionary Biology and Behavior, Michigan State University, East Lansing, Michigan, USA; f Plant Resilience Institute, Michigan State University, East Lansing, Michigan, USA; Oregon State University

**Keywords:** antibiotic resistance genes, database, isolates, metagenomics, microbial ecology, microbiome dynamics, one health, plasmid, soil microbiology, targeted gene assembly

## Abstract

Plasmids harbor transferable genes that contribute to the functional repertoire of microbial communities, yet their contributions to metagenomes are often overlooked. Environmental plasmids have the potential to spread antibiotic resistance to clinical microbial strains. In soils, high microbiome diversity and high variability in plasmid characteristics present a challenge for studying plasmids. To improve the understanding of soil plasmids, we present RefSoil+, a database containing plasmid sequences from 922 soil microorganisms. Soil plasmids were larger than other described plasmids, which is a trait associated with plasmid mobility. There was a weak relationship between chromosome size and plasmid size and no relationship between chromosome size and plasmid number, suggesting that these genomic traits are independent in soil. We used RefSoil+ to inform the distributions of antibiotic resistance genes among soil microorganisms compared to those among nonsoil microorganisms. Soil-associated plasmids, but not chromosomes, had fewer antibiotic resistance genes than other microorganisms. These data suggest that soils may offer limited opportunity for plasmid-mediated transfer of described antibiotic resistance genes. RefSoil+ can serve as a reference for the diversity, composition, and host associations of plasmid-borne functional genes in soil, a utility that will be enhanced as the database expands. Our study improves the understanding of soil plasmids and provides a resource for assessing the dynamics of the genes that they carry, especially genes conferring antibiotic resistances.

**IMPORTANCE** Soil-associated plasmids have the potential to transfer antibiotic resistance genes from environmental to clinical microbial strains, which is a public health concern. A specific resource is needed to aggregate the knowledge of soil plasmid characteristics so that the content, host associations, and dynamics of antibiotic resistance genes can be assessed and then tracked between the environment and the clinic. Here, we present RefSoil+, a database of soil-associated plasmids. RefSoil+ presents a contemporary snapshot of antibiotic resistance genes in soil that can serve as a reference as novel plasmids and transferred antibiotic resistances are discovered. Our study broadens our understanding of plasmids in soil and provides a community resource of important plasmid-associated genes, including antibiotic resistance genes.

## Abstract (precis)

Soil-associated plasmids have the potential to transfer antibiotic resistance genes from environmental to clinical microbial strains, which is a public health concern. A specific resource is needed to aggregate the knowledge of soil plasmid characteristics so that the content, host associations, and dynamics of antibiotic resistance genes can be assessed and then tracked between the environment and the clinic. Here, we present RefSoil+, a database of soil-associated plasmids. RefSoil+ presents a contemporary snapshot of antibiotic resistance genes in soil that can serve as a reference as novel plasmids and transferred antibiotic resistances are discovered. Our study broadens our understanding of plasmids in soil and provides a community resource of important plasmid-associated genes, including antibiotic resistance genes.

## INTRODUCTION

Soil is a unique and ancient environment that harbors immense microbial biodiversity. The soil microbiome has functional consequences for ecosystems, such as supporting plant growth (1, 2) and mediating key biogeochemical transformations (3). It also serves as a reservoir of microbial functional genes of interest to human and animal welfare. Within microbial genomes, important functions can be encoded on both chromosomes and extrachromosomal mobile genetic elements such as plasmids. Plasmids can be laterally transferred among community members, both among and between phyla (4–6). This causes a propagation of plasmid functional genes and allows them to spread among divergent host strains. Within microbial communities, plasmids influence microbial diversification (7) and contribute to functional gene pools (4). Plasmids can alter the fitness of individuals in a community as they can be gained or lost in the environment, which alters their functional gene content and can have consequences for their local competitiveness.

Antibiotic resistance genes (ARGs) provide a prime example of the importance that functional genes encoded on plasmids can have. ARGs can undergo plasmid-mediated horizontal gene transfer (HGT) (8, 9). There is particular concern about the potential for spread of ARGs between environmental and clinically relevant bacterial strains. Studies of ARGs in soil have shown overlap between environmental and clinical strains that suggests HGT (10–12). For example, plasmid-encoded quinolone resistance (*qnrA*) in clinical *Enterobacteriaceae* strains likely originated from the environmental strain Shewanella algae (11). The extent of the impact of environmental reservoirs of ARGs is unknown (13), but studies have shown evidence for predominantly vertical, rather than horizontal, transfer of these genes (14). Additionally, it is speculated that rates of transfer in bulk soil are low compared to that in environments with higher population densities, such as the rhizosphere, phyllosphere, and gut microbiomes of soil microorganisms (15). In the case of antibiotic resistance, mobilization is a public health risk. Broadly, the ability of plasmids to rapidly move genes both between and among memberships is linked to diversification in complex systems, especially soils (7).

Despite their ecological and functional relevance, plasmids are not well characterized in soil. Plasmids vary in copy number, host range, transfer potential, and genetic makeup (4, 16), making them difficult to assemble and characterize from complex soil metagenomes that contain tens of thousands of bacteria and archaea (17). Plasmid extraction from soil is biased toward smaller plasmids and excludes linear plasmids (4). Additionally, mosaic gene content on plasmids makes their assembly from metagenomes difficult (4). Though new methods for plasmid assembly from metagenomes are being developed (18, 19), the resulting contigs represent a population average of plasmid gene content and size because they are very likely not derived from an individual cell. Thus, the size ranges of plasmids in soils are largely unknown but of consequence, because size is one factor reported to contribute to plasmid potential for transferability (5). Furthermore, “plasmidome” analysis and plasmid assembly from metagenomes do not provide host information. New methods, such as single-cell analysis and proximity ligation of chromosomes to plasmids prior to sequencing (20), are still expected to assemble plasmids with some degree of mosaicism. However, whole genomes sequenced from soil-associated microorganisms, inclusive of both chromosomes and plasmids, could provide plasmid host and size information. A database including this information could also provide information as to the extent functional genes encoded on plasmids overlap with the host cell chromosome(s).

To aid in the study of plasmids and their associated functional genes in soil, we established a resource to compare genetic locations of functional genes in soil microorganisms. We extended the RefSoil database (21) of 922 soil microorganisms to include their plasmids. We used this database to test whether soil-associated plasmids are distinct from plasmids from a broad general database of microorganisms, RefSeq (22). We focused our comparisons on plasmid size and the content, diversity, and location of ARGs on plasmids and chromosomes. We used hidden Markov models from the ResFams database (23) to search for ARGs in the extended soil database, RefSoil+, and RefSeq. RefSoil+ provides insights into the range of plasmid sizes and their functional potential within soil microorganisms. RefSoil+ can be used to inform and test hypotheses about the traits, functional gene content, and spread of soil-associated plasmids and can serve as a reference for plasmid assembly from metagenomes.

## RESULTS AND DISCUSSION

### Plasmid characterization.

**FIG 1:**
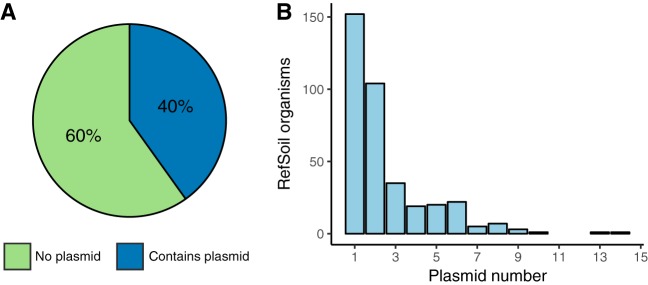
Summary of RefSoil plasmids. (A) Percentages of RefSoil microorganisms with (blue) and without (green) detected plasmids. (B) Distribution of the number of plasmids per RefSoil microorganism.

RefSoil+ is an extension of the RefSoil database inclusive of soil-associated plasmids. RefSoil+ includes taxonomic information, amino acid sequences, coding nucleotide sequences, and GenBank files for a curated set of 922 soil-associated microorganisms. A total of 928 plasmids were associated with RefSoil microorganisms, and 370 RefSoil microorganisms (40.1%) had at least one plasmid (Fig. 1A). This is high compared to the proportion of noneukaryotic plasmids in the general RefSeq database (34%; Mann-Whitney U, *P* < 0.01). The mean number of plasmids per RefSoil organism was 1.01, but the number of plasmids per organism varied greatly (variance, 3.2) (Fig. 1B). For example, strain Bacillus thuringiensis serovar *thuringiensis* (RefSoil 738) had 14 plasmids, ranging from 6,880 to 328,151 bp. The mean number of plasmids per RefSoil organism was also greater than for RefSeq (Mann-Whitney U, *P* < 0.01). The abundance of plasmids found in RefSoil genomes highlights plasmids as an important component of soil microbiomes (7, 24).

**FIG 2:**
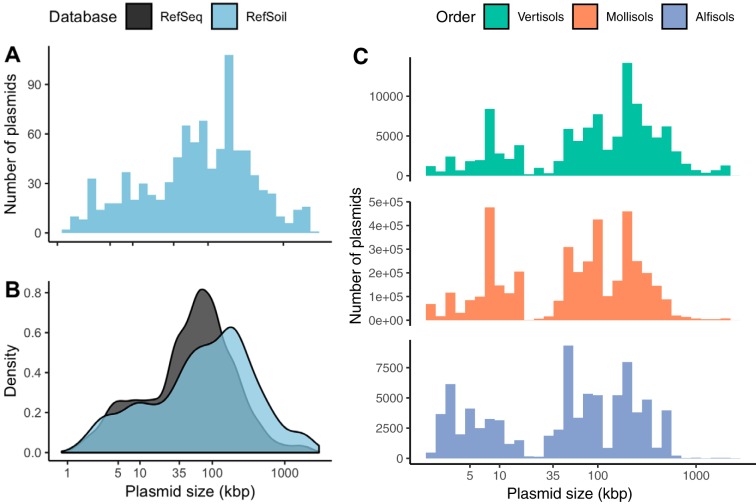
Plasmid size distributions. (A) Histogram of plasmid size (kbp) from RefSoil plasmids. (B) RefSoil (blue) and RefSeq (gray) plasmid size distributions. (C) Estimation of plasmid size distribution in three soil orders. Color indicates soil order and the number indicates the community size.

Soil-associated plasmids tended to be larger than plasmids from other environments (Mann-Whitney U, *P* < 0.01). Plasmid size in RefSoil microorganisms ranged from 1,286 bp to 2.58 Mbp (Fig. 2A), which rivals the range of all known plasmids from various environments (744 bp to 2.58 Mbp) (16). In the distribution of plasmid size, both upper and lower extremes had representatives from soil. Plasmids from all habitats were previously shown to have a characteristic bimodal size distribution with peaks at 5 kb and 35 kb (15–17). In this analysis, the subset RefSeq plasmids had a multimodal distribution (Hartigans’ dip test, *P* < 0.01; bimodality coefficient, 0.745) and modes at 3 kb and 59 kb (Fig. 2). Soil-associated plasmids in RefSoil+ also had a multimodal size distribution (Hartigans’ dip test, *P* < 0.05; bimodality coefficient, 0.800) but had modes at 1 kb, 3 kb, 49 kb, and 183 kb. Additionally, RefSoil+ plasmids were larger than RefSeq plasmids (Mann Whitney U, *P* < 0.01) (Fig. 2). Specifically, RefSoil+ proportionally contained more plasmids of >100 kb (Fig. 2B). Thus, while soil-associated plasmids vary in size, they are, on average, large. This is of particular importance because of the established differences in mobility of plasmids in different size ranges (5). Smillie and colleagues showed that mobilizable plasmids, which have relaxases, tend to be larger than nontransmissible plasmids, with median values of 35 and 11 kbp, respectively (5). The majority of soil-associated plasmids (68.2%) were >35 kbp (Fig. 2), suggesting they are more likely to be mobile. Additionally, conjugative plasmids, which encode type IV coupling proteins, have a larger median size (181 kbp) (5). Similarly, RefSoil+ plasmids had a mode of 183 kb (Fig. 2), suggesting that these soil-associated plasmids are more likely to be conjugative. Future works should examine the genetic potential for the transfer of plasmids associated with different ecosystems to test this hypothesis.

Plasmid size may vary in the environment. To estimate the environmental size distributions of plasmids, we used estimates of the environmental abundance of RefSoil microorganisms (21). We focused on soil orders previously shown to include the most RefSoil representatives (alfisols, mollisols, and vertisols) (21). We found that plasmid size distributions varied based on soil order (Kruskal-Wallis, *P* < 0.01) (Fig. 2C). True environmental abundance may vary based on plasmid copy number within individuals and plasmids from uncultivated microorganisms, but this estimation gives a rough idea of plasmid size distributions in the environment and provides some baseline information because there are methodological challenges to accurately measuring plasmid size *in situ* (4, 18, 19).

**FIG 3:**
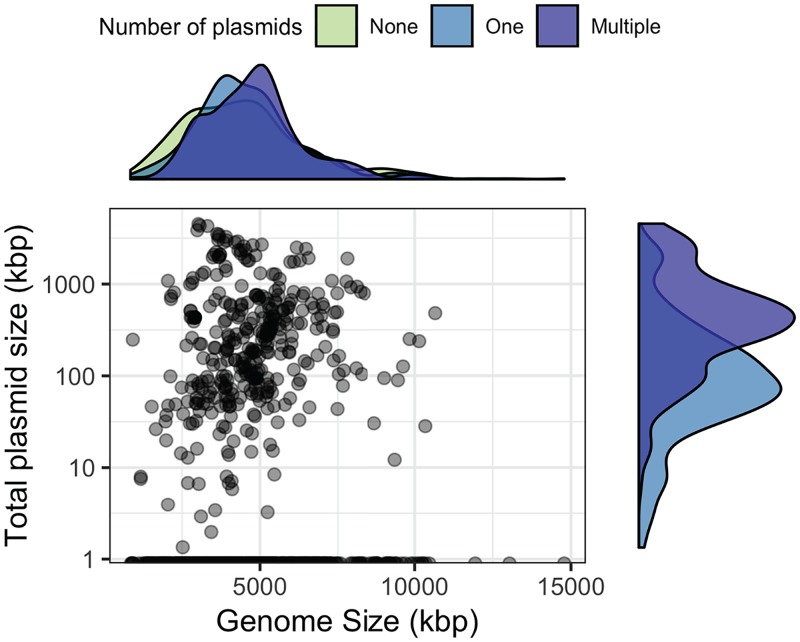
Relationship between plasmid size and genome size. Total plasmid size (sum of all plasmids in a microorganism; kbp) is plotted on a log scale against total genome size for each RefSoil microorganism. Density plots are included for each axis to represent the distribution of RefSoil microorganisms with different numbers of plasmids (none, green; one, blue; or multiple, purple).

Genome size, inclusive of chromosomes and plasmids, is an important ecological trait that is difficult to estimate from metagenomes (25). Due to incomplete assemblies, genome size must be approximated based on the estimated number of individuals through single-copy gene abundance (26). Extrachromosomal elements, however, inflate these estimated genome sizes, because they contribute to the sequence information of the metagenome often without contributing single-copy genes (27). While our methodologies do not account for plasmid copy number (28), we examined the relationship between genome size and plasmid size in soil-associated microorganisms and found a weak but significant correlation (Spearman’s ρ = 0.12; *P *< 0.001) (Fig. 3). Additionally, chromosome size was not predictive of the number of plasmids (Fig. 3; see also Fig. S1 in the supplemental material). For example, Bacillus thuringiensis serovar *thuringiensis* strain IS5056 had the most plasmids in RefSoil+, but these plasmids spanned the size range of 6.8 to 328 kbp. This strain’s plasmids make up 19% of its coding sequences (29), but its chromosome (5.4 Mbp) is average for soils (27). Despite the weak relationship between genome size and plasmid characteristics within these data, the plasmid database can be used to inform estimates of average genome sizes from close relatives detected within metagenomes.

10.1128/mSystems.00349-18.1 FIG S1 Relationship between plasmid number and chromosome size. Boxplots showing the distribution of genome sizes based on the number of plasmids. Numbers above boxplots show the numbers of microorganisms in that category. *P* value from an ANOVA is also shown. Download FIG S1, EPS file, 0.4 MB. Copyright © 2019 Dunivin et al. 2019 Dunivin et al. This content is distributed under the terms of the Creative Commons Attribution 4.0 International license.

### ARGs on soil plasmids.

**FIG 4:**
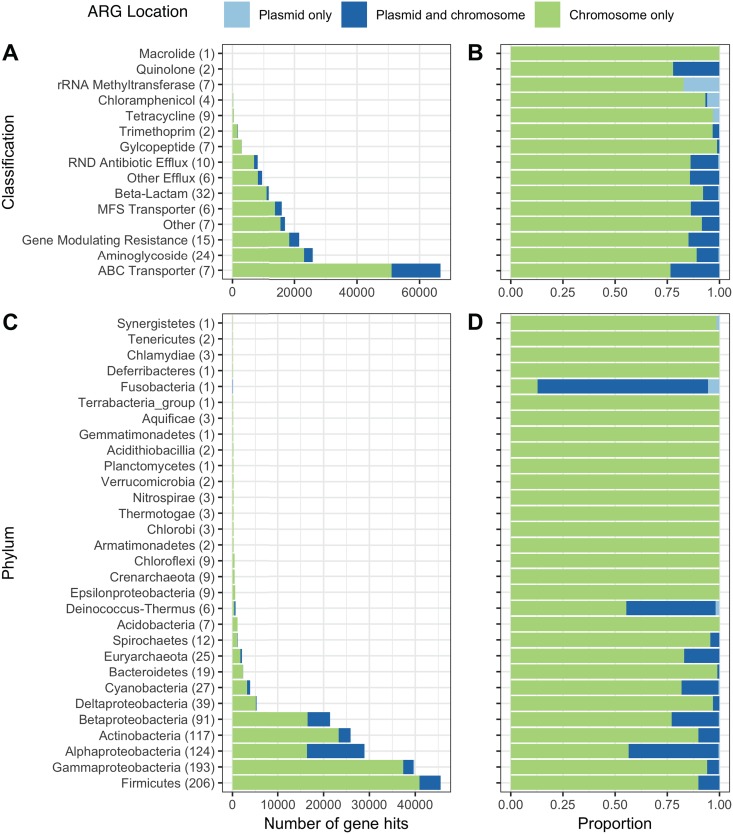
Distribution of ARGs in RefSoil genomes and plasmids. The raw numbers (A) and proportions (B) of ARGs on plasmids (light blue), genomes (green), or both (dark blue) in RefSoil+ microorganisms by antibiotic resistance gene group. The numbers of genes included in each group are shown in parentheses. The raw numbers (C) and proportions (D) of detected ARGs on plasmids (light blue), genomes (green), or both (dark blue) in RefSoil+ microorganisms by phylum-level taxonomy. The numbers of taxa included in each phylum are shown in parentheses.

It is unclear whether soil ARGs are predominantly on chromosomes or mobile genetic elements. While mobile gene pools are not static, there is evidence to suggest low transfer of ARGs in soil (14, 15, 30). For example, bulk soils are not a “hot spot” for HGT because they are often resource-limited (31), and surveys of ARGs in soil metagenomes have suggested a predominance of vertical transfer, rather than horizontal transfer, of ARGs (14, 30). Using RefSoil+ sequences and ResFams hidden Markov models (HMMs) (23), we examined 174 genes encoding resistance to beta-lactams, tetracyclines, aminoglycosides, chloramphenicol, glycopeptides, macrolides, quinolones, and trimethoprim. After quality filtering, we detected 154,392 ARG sequences in RefSoil chromosomes and plasmids (Fig. 4; see also Table S1).

10.1128/mSystems.00349-18.4 TABLE S1 Quality filtered antibiotic resistance gene hits in RefSoil genomes and plasmids; information on quality scores and accession numbers for each ARG hit. Download Table S1, CSV file, 14.1 MB. Copyright © 2019 Dunivin et al. 2019 Dunivin et al. This content is distributed under the terms of the Creative Commons Attribution 4.0 International license.

Adding plasmids to the RefSoil database increased the number of functional gene types, or genes that have functional potential (32), represented in the database, as 7 ARGs (16S rRNA methyltransferase, AAC6-Ib, ANT6, CTXM, ErmC, KPC, and TetD) were only detected on plasmids. Notably, these functional genes would be missed if only chromosomes were considered. However, the majority of ARGs were chromosomally encoded in RefSoil+ microorganisms (Fig. 4A and B) (chromosome versus plasmid; Mann Whitney U, *P* < 0.01). We next examined the genomic distributions of ARGs in RefSoil+ based on taxonomy (Fig. 4C and D). Proteobacteria had the most plasmid-associated ARGs, which has been reported previously (33).

**FIG 5:**
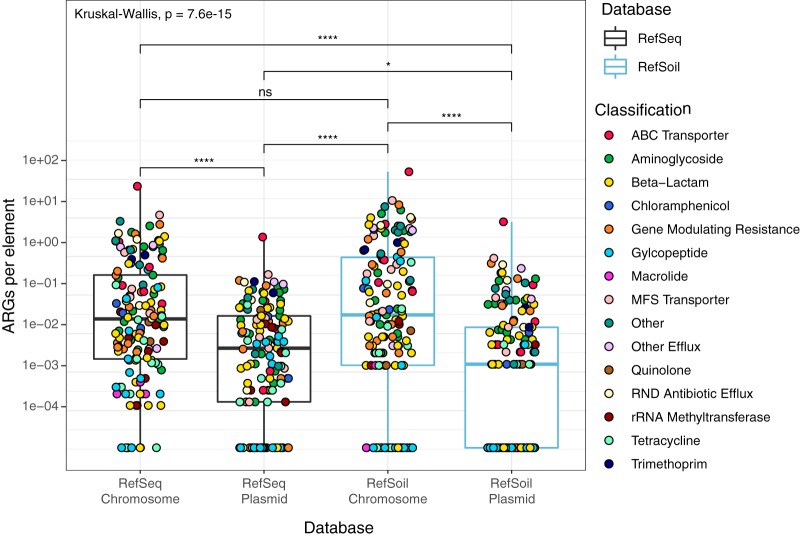
Proportion of ARGs on genomes and plasmids in RefSoil+ and RefSeq databases. Numbers of ARGs were normalized to numbers of genetic elements. Boxplots are colored by database. Points represent individual ARGs and are colored based on classification. Kruskal-Wallis test results are shown in addition to significant results from pairwise Mann-Whitney U tests (****, *P* ≤ 0.0001; *, *P* ≤ 0.05; ns, *P* > 0.05).

We were curious whether ARGs were more commonly detected on chromosomes than plasmids in general or if this trend was specific to soil microorganisms. We found that the number of ARGs per genome was comparable for RefSoil and RefSeq (Mann Whitney U, *P* > 0.05), but RefSoil plasmids had fewer ARGs than RefSeq plasmids (Mann Whitney U, *P* < 0.05) (Fig. 5). Normalizing to individual microorganisms is biased toward chromosomes, however, because chromosomes typically have more base pairs than plasmids. To account for this, we also normalized ARGs to base pairs, and there were more ARGs in plasmids from both databases than in chromosomes (Mann Whitney U, *P* < 0.05). Notably, RefSoil+ had fewer ARGs than RefSeq (Mann Whitney U, *P* < 0.01) (Fig. S3). This suggests that plasmid-mediated HGT rates of ARGs may be relatively low in these soil microorganisms. We note that the RefSoil database is limited in representatives of *Verrucomicrobia* and *Acidobacteria*, which may change these estimates (21); however, this will improve as the database grows.

We examined this trend for each antibiotic class and observed a greater proportion of ARG sequences on plasmids in RefSeq than in RefSoil+ for genes encoding glycopeptide and tetracycline resistance (see Fig. S2). Gibson and colleagues also found a lack of tetracycline resistance genes in soil-associated isolates compared to that in water- and human-associated strains (23). By determining whether ARGs were encoded on plasmids or chromosomes, our analysis suggests that these patterns were due to chromosomal genes and more likely vertically transferred (Fig. 5). Thus, these soil bacteria harbor relatively few ARGs on plasmids, suggesting that RefSoil+ microorganisms have limited capacity for plasmid-mediated transfer of these genes. Future assessments of functional gene content on chromosomes and plasmids together will help to delineate changes in transfer potential and reveal selective or environmental factors that impact transfer potential.

10.1128/mSystems.00349-18.2 FIG S2 Proportion of ARGs by classification in RefSoil and RefSeq databases. Boxplots of the proportion of ARGs per genetic element. Each ARG was normalized to the number of genetic elements in the database. Points are colored by ARG category. Kruskal-Wallis *P* values are shown, and where applicable, significant Mann-Whitney U test results are shown (ns, *P* > 0.05; *, *P* ≤ 0.05; **, *P* ≤ 0.01; ***, *P* ≤ 0.001; ****, *P* ≤ 0.0001). Download FIG S2, EPS file, 1.3 MB. Copyright © 2019 Dunivin et al. 2019 Dunivin et al. This content is distributed under the terms of the Creative Commons Attribution 4.0 International license.

10.1128/mSystems.00349-18.3 FIG S3 Proportion of ARGs on genomes and plasmids in RefSoil+ and RefSeq databases normalized to base pairs. Numbers of ARGs were normalized to total numbers of base pairs. Boxplots are colored by database. Points represent individual ARGs and are colored based on classification. Kruskal-Wallis test results are shown in addition to significant results from pairwise Mann-Whitney U tests. Download FIG S3, EPS file, 1 MB. Copyright © 2019 Dunivin et al. 2019 Dunivin et al. This content is distributed under the terms of the Creative Commons Attribution 4.0 International license.

While genome data from isolates cannot inform on the environmental abundance of ARGs, our data support observations of ARGs in mobile genetic elements in soil from cultivation-independent studies as well. Luo and colleagues observed a low abundance of chloramphenicol, quinolone, and tetracycline resistance genes in soil mobile genetic elements (24), and Xiong and colleagues (34) also observed low abundance of *qnr* genes. Similarly, we observed fewer plasmid-encoded tetracycline resistance genes in soil-associated microorganisms than in RefSeq microorganisms (Fig. S2). We did not observe significant differences for genes encoding quinolone or chloramphenicol resistance; however, these had small sample sizes (*n* = 2 and 3, respectively). Mobile genetic elements in soil have also been shown to have an abundance of genes encoding multidrug efflux pumps and resistance to beta-lactams, aminoglycosides, and glycopeptides (24). Genes encoding beta-lactam and aminoglycoside resistance were comparable between RefSoil+ and RefSeq (Kruskal-Wallis, *P* > 0.05) (Fig. S2). However, plasmid-borne glycopeptide resistance genes were less common in RefSoil+ plasmids (Mann Whitney U, *P* < 0.05).

### RefSoil+ applications.

RefSoil+ is publicly available on GitHub (https://github.com/ShadeLab/RefSoil_plasmids). It includes an excel file linking RefSoil+ organism taxonomy with accession numbers for corresponding chromosomes and plasmids. It also contains several fasta files with coding DNA sequence (CDS) and amino acid sequences. These files can be downloaded directly from GitHub. RefSoil+ has been used to better estimate genome sizes in soil (27) and to estimate the distribution of arsenic resistance genes in soil-associated chromosomes and plasmids (35).

Our results show that soil-associated plasmids have distinctive traits and can harbor functional genes that are not encoded on host chromosomes. RefSoil+ expands the knowledge of functional genes with potential for transfer among soil microorganisms and offers insights into plasmid size and host ranges in soil (and improves the accuracy of estimates of their genome sizes).

Because it is populated by the chromosomes and plasmids of isolates, RefSoil+ links host taxonomy to plasmid content. This linkage is important especially for heterogeneous ecosystems with high microbial richness, such as soils, which rely heavily on cultivation-independent methods for observing microbially diverse populations. RefSoil+ can guide the assembly and support the annotation of plasmids from soil metagenomes and also direct hypotheses of host identity (18, 36). Notably, plasmid gene content is not static (37), and individuals can gain or lose plasmids (38, 39). Despite this, historical data of the genetic makeup and host range of plasmids can be used to better understand plasmid ecology, and to serve as an important reference to understand by how much host plasmid numbers and contents change in the future. This information contributes to information needed to understand patterns of plasmid dissemination, both across environments and among hosts.

RefSoil+ can be used as a reference database or as a database for primer design to target plasmids in the environment. Advances microbiome sequencing methods such as presequencing proximity linkage (e.g., Hi-C [20]), long-read technology (40), or single cell sequencing (41) could add to and leverage RefSoil+ to improve the characterization of plasmid-host relationships in soil. As movements of ARGs are observed in the clinic and the environment, RefSoil+ can also serve as a reference for comparison with legacy plasmid and chromosome contents and distributions. Novel genomes and plasmids could be added in future RefSoil+ versions, and plasmid-host relationships as well as encoded functions could be compared between cultivation-dependent and -independent methodologies. RefSoil+ provides a rich community resource for research frontiers in plasmid ecology and evolution within wild microbiomes.

## MATERIALS AND METHODS

### RefSoil plasmid database generation.

Accession numbers from RefSoil genomes were used to collect assembly accession numbers for all 922 strains. Assembly accession numbers were then used to obtain a list of all genetic elements from the assembly of one strain. Because all RefSoil microorganisms have completed genomes, all plasmids present at the time of sequencing are included in the assembly. Plasmid accession numbers were compiled for each strain and added to the RefSoil database to make RefSoil+ (see Table S1 in the supplemental material). Plasmid accession numbers were used to download amino acid sequences, coding nucleotide sequences, and GenBank files. To ease comparisons between genome and plasmid sequence information, sequence descriptors for plasmid protein sequences were adjusted to mirror the format used for bacterial and archaeal RefSoil files.

### Accessing RefSeq genomes and plasmids.

Complete RefSeq genomes and plasmids were downloaded from NCBI to compare with RefSoil. All RefSeq bacteria and archaea protein sequences were downloaded from release 89 (ftp://ftp.ncbi.nlm.nih.gov/refseq/release). All GenBank files for complete RefSeq assemblies were downloaded from NCBI. A total of 10,270 bacterial and 259 archaeal assemblies were downloaded. GenBank files were used to extract plasmid size and to compile a list of chromosomal and plasmid accession numbers. GenBank information was read into R, and accession numbers for plasmids and chromosomes were separated. Additionally, all RefSoil accession numbers were removed from the RefSeq accession numbers. Ultimately, 10,335 chromosome and 8,271 plasmids were collected to represent non-RefSoil microorganisms. Protein files were downloaded and tidied using the protocol for RefSoil plasmids as described above.

### Plasmid characterization.

We summarized the RefSoil+ and RefSeq plasmids in several ways. Plasmid size was extracted from GenBank files for each RefSoil genome and plasmid. For comparison, size was also extracted from RefSeq plasmids. These data were compiled and analyzed in the R statistical environment for computing (42). The RefSoil metadata (Table S1), which contains host information for each plasmid, was used to calculate proportions of RefSoil microorganisms with plasmids. Both the number of plasmids per organism and the number of RefSoil microorganisms with one plasmid were examined. Plasmid size distributions were compared using Mann Whitney U tests, Hartigan’s dip test (43), and bimodality coefficients (44). The environmental abundances of RefSoil plasmids were calculated using estimations of RefSoil organism environmental abundance (21). Only soil orders with the most RefSoil+ representatives (alfisols, mollisols, and vertisols [21]) were included in the analysis.

### Antibiotic resistance gene detection.

We examined ARGs from the ResFams database (174 total [23] in RefSoil+) (see Table S3). We then used HMMs from the ResFams database (23) to search amino acid sequence data from RefSoil genomes and plasmids with a publicly available custom script and HMMER (45). To perform the search, hmmsearch (45) was used with –cut_ga and –tblout parameters. These steps were repeated for protein sequence data from the complete RefSeq database (accessed 24 July 2018). Tabular outputs from both data sets were analyzed in R. Quality scores and percent alignments were plotted to determine quality cutoff values for each gene (Fig. S1). All final hits were required to be within 10% of the model length and to have a score of at least 30% of the maximum score for that gene. When one amino acid sequence was annotated twice (i.e., for similar genes), the hit with the lower score was discarded. The final quality filtered hits were used to plot the distribution of ARGs in RefSoil genomes and plasmids.

### Data availability.

All data and workflows are publicly available on GitHub (https://github.com/ShadeLab/RefSoil_plasmids). A table of all RefSoil microorganisms with genome and plasmid accession numbers is available in Table S2 and GitHub in the DATABASE_plasmids repository. This repository also hosts amino acid and nucleotide sequences for RefSoil+ genomes and plasmids. Plasmid retrieval workflows are included in the BIN_retrieve_plasmids directory.

10.1128/mSystems.00349-18.5 TABLE S2 RefSoil taxonomy table with plasmid and genome accession numbers. Download Table S2, CSV file, 0.2 MB. Copyright © 2019 Dunivin et al. 2019 Dunivin et al. This content is distributed under the terms of the Creative Commons Attribution 4.0 International license.

10.1128/mSystems.00349-18.6 TABLE S3 ResFams HMMs and antibiotic classifications. Download Table S3, XLSX file, 0.1 MB. Copyright © 2019 Dunivin et al. 2019 Dunivin et al. This content is distributed under the terms of the Creative Commons Attribution 4.0 International license.

All workflows are included on GitHub as well in the ANALYSIS_antibiotic_resistance repository.
